# Organizational and Financial Analysis of Polish Tobacco Control Program in 2000–2018

**DOI:** 10.3390/ijerph17072532

**Published:** 2020-04-07

**Authors:** Łukasz Balwicki, Anna Tyrańska-Fobke, Małgorzata Balwicka-Szczyrba, Marlena Robakowska, Michal Stoklosa

**Affiliations:** 1 Department of Public Health & Social Medicine, Medical University of Gdańsk, 42A Zwycięstwa St., Gdańsk 80-210, Poland; mrobakowska@gumed.edu.pl; 2 Department of Emergency Medicine, Medical University of Gdańsk, 17 Smoluchowskiego St., Gdańsk 80-214, Poland; 3 Department of Commercial Law, University of Gdańsk, 6 Bazynskiego St., Gdańsk 80-309, Poland; m.balwicka@prawo.ug.edu.pl; 4 American Cancer Society, 250 Williams St., Atlanta, GA 30303, USA; michal.stoklosa@cancer.org

**Keywords:** tobacco, smoking, community health, tobacco policy, program efficiency, Poland

## Abstract

In accordance with the provisions of the WHO Framework Convention on Tobacco Control (FCTC), each country shall promote and strengthen public awareness of tobacco control issues (Article 12). Many parties to the FCTC have adopted national tobacco control programs to organize their tobacco control activities. The aim of our study was to analyze the organization and funding of the Polish Tobacco Control Program in years 2000–2018. Document analysis of The Program and reports from its implementation were performed in accordance to the Agency for Health Technology Assessment in Poland (AHTAPol) recommendations and the WHO FCTC guidelines for Article 12 implementation. Spending was also analyzed. The study showed both inadequate planning of and funding for Polish Tobacco Control Program. The Program was developed without use of best practices detailed in the WHO FCTC guidelines as well as in national guidelines prepared by AHTAPol. The experience of Poland shows that although earmarking tobacco taxes has existed in the law, it has been largely ineffective due to the poor Tobacco Control Program design and insufficient funding resulting from a poor execution of the earmarking law. This may be a warning to other countries to strive to create law, compliance with which can be verified and controlled.

## 1. Introduction

In accordance with the provisions of the WHO Framework Convention on Tobacco Control (FCTC), each country shall promote and strengthen public awareness of tobacco control issues (Article 12). The FCTC lists legislative, executive, administrative and other measures that each country shall adopt and implement in order to reach that goal. In accordance with the requirements of the treaty, many parties to the FCTC have adopted national tobacco control programs to organize their tobacco control activities. This was met with active resistance from the tobacco industry, the overcoming of which required joint efforts by governments with the support of civil society organizations [1,2,3,4].

The guidelines for implementation of Article 12 describe how to design effective educational programs [5]. Other resources on health program planning and creation are also available [6]. Those resources help program leaders in creating goals, choosing the most effective interventions, setting measures, and evaluating activities. 

Beside organizational aspects of any program, one of the most important factors for the program’s success is its funding. Unfortunately, literature providing guidelines regarding proper funding for tobacco control programs is scarce. The FCTC Guidelines do not indicate the amount of funding sufficient to ensure program effectiveness. According to the Centers for Disease Control and Prevention (CDC), spending on tobacco programs should be: 3.99 USD to 6.75 USD per person, per year for community interventions, 0.65 USD to 1.95 USD per person, per year for mass-reach health communication interventions, 2.04 USD to 5.94 USD per adult, per year for cessation interventions, 10% of total program budget for surveillance and evaluation, and 5% of total program budget, or the cost of 25% to 100% of a full-time equivalent dedicated staff person, whichever is greater, for infrastructure, administration, and management [7]. These guidelines are, however, U.S.-specific. In 2011, the WHO estimated that low/lower-middle-income and upper-middle-income countries needed 0.10 USD–0.23 USD and 0.11 USD–0.72 USD per capita, respectively, in order to control the demand for tobacco [8]. The funds available in those countries were, however, vastly inadequate [9,10].

In Poland, tobacco control program activities were based on the provisions described in the Act on the Protection of Public Health against the Effects of Tobacco Use of 9 November 1995 [11]. This act introduced the Tobacco Control Program in 1997 (The Program for Reducing Health Consequences of Tobacco Smoking, in Polish: Program Ograniczania Zdrowotnych Następstw Palenia Tytoniu). The Program was set out in the original act but the funding for the Program was not addressed in the law until 1999, when specific funding regulations were enacted and came into force from 1 January 2000. Specifically, the law stated that the program was financed from the state budget at the level of 0.5% of the value of the excise tax on tobacco products (earmarking). This money could be spent directly by the Ministry of Health MoH, its agencies or civil society organizations.

The Act of 27 August 2004 on health care benefits financed from public funds (Article 5), defines a health policy program as a set of planned and intentional healthcare interventions, designed, implemented, conducted and financed by the Minister or a local government agency, that is described as effective, safe and expected to achieve, in a designated time frame, goals of detecting and meeting healthcare needs and improving the health of a defined group of beneficiaries. According to this law, Ministers and local government agencies may design, implement, conduct and finance health policy programs (HPP). Another article (Article 48a) provides a detailed list of components required of an HPP and points to the procedures and criteria for assessing such programs by the Polish Agency for Health Technology Assessment and Tariff System (AHTAPol) [12].

Globally, the economic cost of smoking in terms of health care-related expenses and the lost productivity of those sickened or killed by tobacco amounts to nearly 2 trillion USD (in 2016 PPP) each year, equivalent to almost 2% of the world’s total economic output [12]. In Poland, this cost amounts to 32 billion USD per year [13]. Successful Tobacco Control Program implementation would lead to a lower prevalence of tobacco use and, hence, reduce the health and economic burden of tobacco. In fact, it has been established that tobacco control interventions are among the “best buy” interventions in public health [14].

On the one hand, Poland has had huge successes in reducing tobacco smoking. Data show that smoking prevalence in Poland declined between 1976 and 2014 from 73% to 28% among men and from 30% to 19% among women [15]. During this time, several measures were implemented including the enactment of the Tobacco Control Act in 1995 with following revisions, as well as tobacco tax increases, which incorporated, to some extent, many of the proven measures to reduce tobacco use worldwide defined in WHO MPOWER package [16]. On the other hand, not much progress has been observed in the most recent years, with some observed increases in the use of electronic cigarettes among teenagers (an increase from 5.5% in 2010 to 29.9% in 2014) [17]. The aim of our study was to analyze the organization and funding of the Polish Tobacco Control Program in the years 2000 to 2018.

## 2. Materials and Methods

We have analyzed annual reports from the implementation of The Program for Reducing Health Consequences of Tobacco Smoking. Since 1997, there have been five editions of The Program. Additionally, the most recent Program for the years 2014–2018 [18] was subjected to a detailed analysis as a program that should be of the highest quality due to the experience of implementing earlier activities and availability of guidelines.

We have compared the Program with available national and international guidelines to see if the Program meets their criteria. We checked whether the key elements of the program were described and whether the descriptions were precise. The guidelines were: guidelines for effective health programs prepared by AHTAPol in 2010 and WHO FCTC Guidelines for Article 12.

AHTAPol has created the aforementioned model for health policy programs (HPP) to help central and local public institutions create effective health programs. It is an evidence-based model that emphasizes setting goals and measures of effectiveness [19]. It was designed on the basis of health assessment guidelines and the European Network for Health Technology Assessment (EUnetHTA) recommendations [20]. Particular attention was paid to the presence and precise description of all recommended components of a Program:

- Description of the background;
- Description of the health problem;
- Program goals—description of the general goal, description of specific goals, whether the goals meet the SMART (specific, measurable, achievable, relevant and time-bound) criteria [21], and whether there are expected outcomes and measures of effectiveness relevant to the goals;
- Description of the beneficiaries of the Program;
- Program design—is the Program divided into phases of implementation, are the planned interventions clearly defined including the time frames of completion, is there any evidence for effectiveness of the interventions undertaken as part of the Program, are there EBM-based guidelines or standards of managing the defined healthcare problem;
- Costs/budget;
- Arguments regarding optimal usage of available resources;
- Description of monitoring and evaluation of the Program.

Apart from the AHTAPol recommendations, the WHO FCTC guidelines for Article 12 implementation present a checklist of elements that should be included in national tobacco control programs to ensure their effectiveness. The checklist includes: vision and mission statement, goals and objectives, strategies and expected results for each objective, budget, entities responsible for each activity, target dates and required resources, progress indicators, monitoring and evaluation of progress and outcomes, result dissemination. 

In addition to comparing the Program to available guidelines, we analyzed spending on the Program in years 2000–2018. We obtained data on funding from the annual reports on the implementation of the Program. We calculated the total spending for each year, and compared it with the sums related to the provisions described in the law (0.5% of tobacco excise tax). We also calculated the amount of money that should have been allocated to the the Program. Due to lack of relevant data, we could not analyze the effectiveness of the Program. Reports from the implementation of the Program did not contain information on whether the goals of the Program were achieved through the reported activities. There was also no data on the established measures of effectiveness.

## 3. Results

The Program included the following tasks: monitoring the situation in the field of use and cultivation of crop tobacco, effective protection from tobacco smoke, offering assistance in smoking cessation, information and warnings about the risks, health issues related to the use of tobacco, eliminating marketing practices violating the prohibition of advertising and promotion of tobacco, implementing economic and administrative stimuli limiting the consumption of tobacco products.

### 3.1. Organization of the Program

Table 1 presents analysis of the Program on Reducing Health Consequences of Tobacco Smoking aims for years 2014–2018 in comparison with AHTAPol guidelines. Specifically, we analyzed if the document met the requirements of an effective health policy program.

We identified several instances in which the description of activities included in the Program were imprecise. The eligible population was not clearly defined, and simply stated that the Program was addressed to ‘‘the Polish society”. What is more, the detailed objectives of the Program did not precisely specify the effectiveness measures, and no initial and final values for the effectiveness measures were indicated, so no SMART criteria were applied. The tasks described in the document were not designed as the stages of Program implementation and had no deadlines. The entire document did not mention evaluation and monitoring. Only the requirement for the preparation of annual reports on the implementation of the tasks was listed. It is worth stating that in the last available annual report on the Program’s implementation from 2016, the following summary of the implemented activities can be found: “Due to the limited budget resources that could be used to finance Program tasks, their implementation in 2016 focused mainly on priority tasks, i.e., on the monitoring, educational and information activities directed to the people exposed to passive smoking, as well as on conducting education among various target groups” [22].

The specific objectives of the Program are described for preventive measures (e.g., Preventing an increase in the number of people who start smoking, preventing an increase in exposure to tobacco smoke in public places) and actions from the level of intervention (e.g., Creating appropriate legal regulations enabling the implementation of an effective policy for limiting tobacco use in Poland; increasing the knowledge about the harmfulness of tobacco smoking among children and adolescents; a change in attitudes towards smoking, aimed at marginalizing this phenomenon in society; increasing the number of people who quit smoking). For none of these specific objectives measurement methods or criteria were identified. 

When compared to the FCTC Article 12 guidelines (Table 2), the Tobacco Control Program was missing monitoring and evaluation of outcomes and dissemination of results to people, bodies or entities responsible for tobacco-control education, communication and training. What is more, the Polish Program strategies were chosen for tasks and not for goals, the expected results were not specified, only their indicators. The budget plan was not prepared. The document contained only a description of the sources of financing for specific tasks in the Program, but not the monetary value. Additionally, the document neither stated the vision nor provided a mission statement. 

### 3.2. Financial Analysis

As no Program budget was planned and published ahead, we could not analyze spending on particular Program activities. Therefore, we concentrated our analysis on the comparison of spending on the Program with data on the portion of tobacco tax revenue that should have been spent on the Program according to the law, i.e., the 0.5% of the total tobacco tax revenue. The figures are presented in Table 3. The average annual legislated spending on the Program was 21 million USD. However, the average annual actual expenditure amounted to 0.6 million USD, which was only 3% of the funds due. In the years 2000–2017, the average annual actual level of funding amounted to only 0.026% of total tobacco excise tax revenue, as opposed to the 0.5% assumed stipulated by the tobacco control law. What is more, the financial gap accumulated over the years. The total amount of funds missing in financing the Polish Tobacco Program was about 368 million USD for the years 2000–2017. One of the main reasons for the inadequate funding of the Program was that the law did not describe the way revenues from excise tax ought to be directed to the Ministry of Health, its agencies or civil society organizations. The figure of 0.5% of tobacco excise tax revenue served only as a guideline value for the Ministry of Health on how much money they should give to the Program. Only once, in 2000, the MoF added an extra 21 million Polish złoty (PLN) to the MoH budget for the new task, which was the Program itself. The interpretation of the law expressed by the Ministry of Finance was: the Minister of Health should allocate in his/her budget (that is a part of the overall state budget) the amount of funds equivalent to 0.5% of the revenues from the tobacco excise tax to the Program. It was the MoH’s responsibility to allocate the relevant funds to the Program in the ministry budget. Historical records show that the MoH never fulfilled this obligation [24]. There were no consequences (administrative or personal) for this failure. Experts believe that there was never a real political will to fund the Program [25].

## 4. Discussion

Our study showed both inadequate planning of and funding for the Polish Tobacco Control Program. The Program was developed without the use of best practices detailed in the WHO FCTC guidelines as well as in national guidelines prepared by AHTAPol. There can be several explanations for these situations. The lack of proper planning might be due to shortages of staff dedicated to the coordination of tobacco control activities. The preparation of the Program is the responsibility of the Ministry of Health, which has an agency for its execution (Chief Sanitary Inspectorate). However, within the Ministry, the Department of Public Health, which is responsible for tobacco control, has a very limited number of positions devoted specifically to tobacco control–this may also be a reason for the faults in the development of the Program. This situation could have been handled with the help of external experts from health agencies like the National Institute of Public Health, as well as from civil society organizations, which were very active at that time. To the authors knowledge, no cooperation was established with these institutions on planning the Program’s editions. Moreover, for several years, Poland was supported by the Bloomberg Initiative grants and many activists were sponsored to attend prestigious Global Tobacco Control Leadership Program in USA. However, it appears that the MoH never attempted to utilize this knowledge within the tobacco control programs. The lack of proper planning and execution of the Program was also noticed by the Polish Supreme Audit Office. In its report from 2013, the Office clearly stated that the Chief Sanitary Inspectorate, responsible for leading the Program from 2006 onwards, had never conducted a reliable monitoring and evaluation of the effects of its implementation. In general, a methodology allowing for an assessment of the implementation of the strategic aim and most of the detailed aims has never been developed: “The objectives of the Tobacco Control Program, which defined the directions of activities and priority tasks in combating health risks related to smoking, were not achieved, as the conditions for the implementation of the Program were not created: its budget was not separated, reliable monitoring and evaluation of the tasks specified therein was not carried out, and the management and coordination instruments provided for therein were not functioning” [24]. 

The earmarking of tobacco tax for the tobacco control Program did not work either. Spending on tobacco control activities was miniscule compared to what had been agreed in the law, with the mean spending amounting to a mere 3% of what the law stipulated. It may seem that the Program was never prioritized, and that the support from the Bloomberg Initiative, which financed grants for the Chief Sanitary Inspectorate and a few civil society organization between 2007–2013 [26] not only did not change Polish Government position, which continued with its decision not to finance tobacco control activity from the state budget, but in fact resulted in crowding out the public by private financing [9].

Although Poland achieved a significant reduction in daily smoking prevalence among people aged 15+ from 33% in 1999 to 24% in 2015 [27], there is no in-depth scientific analysis on the impact of the Program itself, especially its parts where funding is required: monitoring the situation, educational campaigns, smoking cessation services. The report of the Supreme Audit Office [24] states that the Program that should have been funded from the earmarked tax did not result in changes in smoking rates [28]. The Office also pointed out that they could not assess the Program as there was no reliable monitoring and evaluation of the aims and activities, and there was a lack of proper management and coordination. It was mainly due to the lack of proper funding as the way of allocating money was neither described in the law nor in the Program itself. The Ministry of Finance refused to transfer money legally due to the program to the MoH’s budget, and suggested the MoH finds the funds in its own budget, which had a profoundly negative effect on the Program [29]. At the same time, the MoH was not compelled to find the money in the MoH budget as there was always competition with other health programs [28]. There are several possible reasons for the progress in tobacco use reduction. First, the strong involvement of tobacco control community and, among them, leaders who pushed for effective tobacco control legislation (smoke-free laws) [30]. Next, the support from the Bloomberg Initiative which strengthened non-governmental organizations, which started to advocate for better tobacco controls. Such support for countries with limited resources for tobacco control show that a transnational tobacco control network can be very helpful in developing effective tobacco control measures [3,31,32]. Finally, after Poland joined the European Union (EU) in 2004, the country was obliged to meet the EU minimums for tobacco taxes [33]. Those EU-induced tax hikes led to significant increases in cigarette prices, which drove cigarette consumption down [34].

One of the biggest advantages of well-implemented tobacco control programs is their high cost-effectiveness. The cost of a life year saved through tobacco control is very low compared to other public health interventions. For example, it has been estimated that the Tips from Former Smokers, an anti-smoking media campaign prepared by the Centers for Disease Control and Prevention (CDC) cost only 393 USD per life year saved in the U.S., which by WHO standards makes the intervention very cost-effective [35]. Unlike many other health interventions, tobacco control does not require medical procedures, which drives the costs of the intervention down. Table 4 compares the cost of Tobacco Control Program to some other health interventions that require medical procedures in Poland. 

The analysis presented in this paper has several limitations. Due to the inconsistency in reporting as well as the lack of indicators of success we could not analyze the specific results of the Program. Analysis of reported activities did not allow us to draw any conclusions on the effectiveness of the Program. We could also not evaluate spending for the Program as no budget was planned ahead.

## 5. Conclusions

Globally, countries do not give sufficient priority to tobacco control and Poland is a case in point. It seems necessary to put pressure on governments with the help of national and international NGOs. This activity can balance internal inertia and stop the negative impact of the tobacco industry. Governments should therefore support such organizations as they are their allies in the battle for public interest. They should also use experts in planning health programs and ensure legally sustainable funding for preventive activities. Otherwise, governments will always find “other urgent needs” worth funding. 

Creating overly general legal regulations, which omit the issues of verification/control of the implementation of statutory obligations, is another reason for the discussed failure of the Program’s financing. Legal obligations should be formulated with a clear and specific scope, as only in this way a law that is respected will be created. In such an important area of health protection, legal regulations should not give state authorities too much freedom in implementing the statutory obligations. The example of Poland may be a warning to other countries to strive to create law, compliance with which can be verified and controlled, i.e., certain entities have the competence to do so and certain entities are interested in proper law enforcement.

Guidelines for the implementation of WHO FCTC Article 6 call countries to dedicate a portion of tobacco tax revenues to tobacco control programs. According to the latest WHO report on the global tobacco epidemic 2019, only a handful of countries do so. The experience of Poland shows that although earmarking of tobacco tax was provided for by the law, it has been largely ineffective due to the poor design of the Tobacco Control Program and insufficient funding resulting from a poor execution of the earmarking law.

## Figures and Tables

**Table 1:** Comparison of the content of the Polish Tobacco Control Program for years 2014–2018 with the AHTAPol guidelines–model health policy program (HPP).

Model HPP Criteria		Polish Tobacco Control Program
Background	program time frame	described
	authors	described
	continuation/ program longevity	described
Description of the health problem	definition	described imprecisely
	epidemiology	described
	qualified population	described imprecisely
	current situation	**not described ^1^**
	justification for implementation	described
Program goals	general goal	described imprecisely
	specific goals	described not using the SMART criteria
	effects	**not described**
	measures of effectiveness	described imprecisely
Beneficiaries of the program	population assessment	**not described**
	recruitment into the program	**not described**
Program design	stages	**not described**
	planned interventions	described
	qualification criteria	**not described**
	evidence of program effectiveness	**not described**
	standards, guidelines	**not described**
Costs	cost per unit	**not described**
	estimated total costs	**not described**
	funding sources	described imprecisely
Arguments regarding available resources	reasons for choosing the program	**not described**
Monitoring and evaluation	assessment of recruitment	**not described**
	assessment of service quality	**not described**
	assessment of effectiveness	**not described**
	assessment of outcomes’ durability	**not described**

^1^ In bold criteria which were not described.

**Table 2:** Analysis of implementation of the Guidelines for The Implementation of Article 12 Of the WHO Framework Convention on Tobacco Control (Education, Communication, Training and Public Awareness) in a Polish Tobacco Control Program for years 2014–2018.

Guidelines for the Implementation of Article 12 of FCTC (Education, Communication, Training and Public Awareness) [23]	Polish Tobacco Control Program 2014–2018
Indicative (non-exhaustive) checklist for an action plan for the implementation of education, communication and training activities within a comprehensive tobacco-control program	
State the vision	**No ^1^**
Develop a mission statement	**No**
Formulate goals and objectives	Yes
Select strategies and expected results for each objective	**No**
Prepare a budget plan	**No**
Indicate who is responsible for each activity	Yes
Set target dates and determine the resources required	**No**
Identify progress indicators to enable measurement of the effectiveness of implementation	Indicators identified but not their values
Monitor and evaluate implementation and outcomes	**No**
Disseminate results to people, bodies or entities responsible for tobacco-control education, communication and training	**No**

^1^ In bold criteria which were not described.

**Table 3:** Legislated versus actual spending on the Tobacco control Program in Poland.

Year	Government Revenues from Tobacco Excise Tax ^1^	Legislated Spending on the Program (0.5% of Tobacco Excise Tax Revenue)	Expenditures on Tobacco Control Program ^2^	Actual Expenditure as a Share of Legislated Funds
2000	1 462 355 421 USD	7 311 777 USD	4 831 585 USD	66.08%
2001	1 784 404 675 USD	8 922 023 USD	732 798 USD	8.21%
2002	1 943 181 297 USD	9 715 907 USD	122 564 USD	1.26%
2003	2 174 515 570 USD	10 872 578 USD	128 571 USD	1.18%
2004	2 538 522 332 USD	12 692 612 USD	178 587 USD	1.41%
2005	3 035 652 281 USD	15 178 261 USD	216 397 USD	1.43%
2006	3 625 457 373 USD	18 127 287 USD	521 515 USD	2.88%
2007	4 873 324 177 USD	24 366 621 USD	362 043 USD	1.49%
2008	5 586 958 326 USD	27 934 792 USD	509 360 USD	1.82%
2009	5 153 006 867 USD	25 765 034 USD	405 622 USD	1.57%
2010	5 781 841 695 USD	28 909 208 USD	346 509 USD	1.20%
2011	6 163 258 419 USD	30 816 292 USD	308 946 USD	1.00%
2012	5 704 237 028 USD	28 521 185 USD	310 469 USD	1.09%
2013	5 759 807 644 USD	28 799 038 USD	319 053 USD	1.11%
2014	5 680 551 805 USD	28 402 759 USD	271 554 USD	0.96%
2015	4 718 630 275 USD	23 593 151 USD	193 765 USD	0.82%
2016	4 687 810 606 USD	23 439 053 USD	146 515 USD	0.63%
2017	4 971 977 923 USD	24 859 890 USD	134 574 USD	0.54%
2018	5 485 785 087 USD	27 428 925 USD	no official data	
TOTAL	81 131 278 801 USD	405 656 393 USD	10 040 427 USD	

^1^ Data from reports on state budget implementation, Ministry of Finance; ^2^ data from annual reports on the Program for Reducing Health Consequences of Tobacco Smoking implementation.

**Table 4:** Comparison of the funds for selected health interventions in Poland (2013) ^1^.

Type of Expenditure	Amount
Total expenditure for health from state budget	1,365,663,134 USD
Expenditure for health policy programs from state budget	279,250,672 USD
Expenditure for Tobacco Control Program	318,983 USD
Colon cancer screening program (excluding medical services)	5,675,153 USD
Primary cancer prevention program	569,350 USD
Cervical cancer and breast cancer screening program (excluding medical services)	6,419,314 USD

^1^ Data from the Report on the state budget implementation in 2013, Report on National Cancer Disease Control Program implementation in 2013.
